# A prospective cohort observational study to validate a simplified postoperative nausea and vomiting severity scale and its effects on sleep and vitality

**DOI:** 10.1186/s12871-025-03074-2

**Published:** 2025-05-10

**Authors:** Shih-Feng Weng, Yu-Hwa Wu, Tin-Wei Kang, Chia-Chih Alex Tseng

**Affiliations:** 1 Department of Healthcare Administration and Medical Informatics, Kaohsiung Medical University, Kaohsiung, Taiwan R.O.C.; 2 Department of Biomedical Engineering, National Cheng Kung University, Tainan, Taiwan R.O.C.; 3 Department of Anaesthesiology, College of Medicine, National Cheng Kung University, No.138, Sheng Li Road, Tainan, 704 Taiwan R.O.C.; 4 Department of Anaesthesiology, Kaohsiung Show Chwan Memorial Hospital, Kaohsiung, Taiwan R.O.C.

**Keywords:** PONV, Severity, Antiemesis, Sleep, Vitality, SPONVSS score

## Abstract

**Background:**

Validating postoperative nausea and vomiting (PONV) severity based on antiemetics prescription can help actively manage PONV by the Rhodes Index of Nausea, Vomiting, and Retching (RINVR) scale but is relatively complicated. This study aims to validate the RINVR scale on PONV intensity with prescribed antiemetics usage, and further simplify and validate this scale to serve as the simplified PONV severity score (SPONVSS) and its effects on sleep quality and vitality.

**Methods:**

This study analyzed data from patients who underwent anesthesia with sleep and vitality reports after surgery. We assessed the PONV severity by RINVR. We also simplified the RINVR score with one element selected from each dimension of PONV. The RINVR score and all 18 simplified combinations were validated with antiemetic usage. These scores were used to study patient-reported sleep quality and vitality.

**Results:**

The AUC of the RINVR score and 18 combinations have a similar high impact on antiemetics administration, sleep, and vitality. Nausea frequency, vomiting, and retching times were chosen as the SPONVSS elements because of overall high AUC and convenience in implementation. Multivariate logistic regression analyses show that besides pain, the SPONVSS score provides a significant impact on postoperative sleep and vitality.

**Conclusions:**

We developed a simple and practical scale to monitor PONV intensity in a broad clinical surgical setting. A high SPONVSS score (≥ 3) is an independent risk for rescue antiemesis, poor sleep quality, and vitality. This scale will be useful to monitor the postoperative care quality and improve postoperative care.

**Supplementary Information:**

The online version contains supplementary material available at 10.1186/s12871-025-03074-2.

## Background

Postoperative nausea and vomiting (PONV) are one of the most common complications after surgical anesthesia. It occurs in approximately one-third of patients who undergo surgery with anesthesia. Patients’ memories of PONV may last over three months and lead to significant consequences [1] and poorer recovery [2–4]. Some guidelines have thus been developed to manage PONV. However, the most commonly used guidelines [5–8] and existing studies focus on the incidences [9–11] instead of the severity of PONV. However, the range of severity is quite large. Some patients reported adequate comfort after vomiting with no treatment needed when visited after operation, whereas some may develop severe PONV that can lead to serious complications, such as an increased risk of organ rupture in rare cases [12]. Furthermore, there have been few reports examining the link between PONV and other aspects of recovery, such as sleep or vitality [13, 14]. This research proposes a scoring system and simplify to 3 factors to evaluate PONV severity in the clinical setting and its effects on sleep and vitality.

According to the current guideline [8, 15], at least two antiemetics were needed by 98.7% of females and 56.8% of male patients with Apfel score ≥ 2, which covers around 80% of the cohort. However, Myles et al. noted only 18% of the patients with PONV had clinically significant symptoms [2]. These conflicting findings can confuse clinicians and patients. They may cause low adherence to guidelines due to lack of awareness, disagreement with the content, routine inertia, and resource limitations such as inadequate staff, time, and finances [16]. In addition, more than 30% of patients at high risk of PONV treated with PONV management guidelines still suffered postoperative emetic symptoms and functional impairment [4]. Therefore, aggressive prescription antiemetics may be necessary for high-risk patients [17]. It is also important to consider what antiemetics have been given for prophylaxis within the last 6 h when prescribing additional antiemetics [8].

Liberal prescription of antiemetics may be a solution, but it is not without side effects especially in some special cases [18]. For the improvement of PONV management with precise antiemetic prescription, it is vital to establish a PONV severity scoring system that patients, caregivers, and doctors can follow. Several severity scoring systems have been used to predict the probability and assess the severity of PONV [1, 19–22]. In 2010, Wengritsky et al. developed a PONV Intensity Scale, validated across a broad patient population, including diverse surgical disciplines. This scale assesses nausea severity, duration, and frequency of retching and vomiting, ensuring its applicability in various clinical settings [3]. This scoring system has been validated to be a reliable tool in different populations [23, 24]. Subsequently, the simplified PONV Impact Scale (SPONVIS) was reported by Myles et al. in 2012 [2]. This Impact Scale relies on nausea severity and the number of vomits or retches in postoperative recovery [2]. In this scale, the cut-off value for clinically important PONV is 5 points. This cut-off point is a bit too strict. The severity of nausea is quite subjective and can lead to differences in assessment. That may not be reflect in Patients’ and caregivers’ perceptions of the need to rescue antiemetics. Thus, we tried to develop another scoring system that can be handled easily and deliver correct information for rescue antiemetics from patients and caregivers at the same time.

This study aims to validate the RINVR scale for PONV severity in relation to rescue antiemetic usage and to develop a simplified PONV severity score (SPONVSS). This new score will focus on predicting antiemetic administration and assessing postoperative sleep quality and vitality.

## Methods

This study was approved by the Ethics Committee of the Ditmanson Medical Foundation, Chia-Yi Christian Hospital (IRB no. 097028), and written informed consent forms were obtained and signed by all subjects. This prospective observational study enrolled 2860 patients who underwent surgery with anesthesia in our hospital from November 2009 to December 2014. After inclusion, 967 patients are enlisted. The inclusion criteria are (1) more than 20 years old; (2) could communicate well; (3) scheduled for surgical procedures under general intubation anesthesia, such as thoracic surgery (chest tubes were inserted in all video-assisted thoracotomy cases), upper abdominal surgery, lower abdominal surgery, gynecological surgery (including laparoscopy), bariatric and other operations (including thyroid cancer surgery, breast surgery, orthopedic surgery, and spine surgery). (4) patients with structured records of sleep quality and vitality. Those who were scheduled for emergency surgery or who could not communicate well were excluded. To ensure the quality of measurement, a maximum of four cases were collected each day during the regular workdays of a week. Vitality refers to the patient’s overall sense of well-being and energy level after surgery, beyond just the presence of pain or nausea. It encompasses how well the patient feels they are recovering, including their sense of having sufficient energy and vigor. Demographic data collected before the operation were age, gender, body weight, height, calculated body mass index (BMI), ASA (American Society of Anesthesiology) score, smoking status, and a history of motion sickness or previous PONV. Pre-operative renal function (i.e. eGFR, creatinine) and the presence of diabetes were also checked for patients before surgery.

### Anesthesia and postoperative data collection

All patients had a face-to-face consultation with the anesthesiologist preoperatively to discuss and confirm the anesthesia plan and preoperative medications. Patients received general anesthesia in different ways, like with or without fentanyl, using propofol (1–2 mg/kg) or sodium thiopentone (3–5 mg/kg), and neuromuscular blockade with atracurium or rocuronium for intubation (reversal with atropine 1.0 mg and neostigmine 2.5 mg). Before anesthesia induction, the patients were not given any preoperative sedative, analgesic, or antiemetic medications. Inhalation anesthetics, including desflurane, isoflurane, or sevoflurane, were used, with no N_2_O administered during anesthesia maintenance. The anesthesia procedures were conducted following standard operating procedures by board-certified anesthesiologists of the authors’ anesthesia team. Patients who received anesthesia were followed up after surgery for 24 h for the use of postoperative rescue antiemetic drugs, including droperidol, dexamethasone, dimenhydrinate, naloxone, metoclopramide, and granisetron. Patient self-reported postoperative outcomes included: (1) PONV score recorded according to RINVR scale with 3 dimensions, including 3 vomiting elements, 2 retch elements, and 3 nausea elements (Table S1). Each element is graded at 0–4 points; (2) sleep quality and vitality state graded as bad, okay, or good in response to the question, “How was your most recent sleep quality or vitality state?” (3) level of pain recorded on a 10-point numeric scale (NRS) when they were at rest, moving or coughing. The patients without coughs were recorded as having no NRS cough and excluded for cough pain analysis. These data were collected independently by two research assistants who were not on the authors’ anesthesia team.

### The simplified RINVR scoring method

For easy clinical practice, we simplified the RINVR scale. The same strategy was used in Myles *et als’* SIPONV and Wengritsky et al.‘s simplified PONV intensity, scale models. The RINVR scale is based on 8 elements that cover 3 dimensions. There are 3 elements related to vomiting (vomiting times, distress, and amount), 3 elements related to nausea (times, severity, and duration), and 2 elements to retching (times and distress). Since our goal is to simplify the RINVR scale down to one element per dimension, 18 different combinations in total need to be compared. The combination that has the higher area under the curve (AUC) with antiemetic administration and quality of sleep and vitality is chosen for our SPONVSS. In our score system, each element is graded 0–4 points, and the resulting total score of the 3-elements scale ranges from 0 to 12. The prediction power of our SPONVSS was further confirmed with the participants’ Apfel score of four PONV risk factors.

### Statistical analysis

Baseline characteristics are presented as mean (± standard deviation) for continued variables and case number (percentage) for categorical variables. Continuous variables were compared using two-sample t-tests, whereas categorical data were analyzed using the χ^2^ test or Fisher’s exact test. Univariate and multivariate logistic regression were used to evaluate the relations between the variables and outcomes, including antiemetic administration, sleep, vitality, and bad sleep and bad vitality (BSV). The variables that are significant in univariate analysis (*p* < 0.05) are further analyzed with multivariate logistic regression. A receiver operating characteristic (ROC) curve was employed to obtain the AUC, which determines the combination of the four outcomes to be used for SPONVSS while the Youden Index was used to get the best cut-off value. The results were presented as odds ratio (OR) with 95% confidence intervals (CIs). All data were analyzed with SPSS 22 (SPSS Inc., Chicago, Illinois).

## Results

A total number of 967 patients were included in this study, with a mean age of 53.7 ± 15.57 years and 68.3% were female. The demographic and postoperative data are presented in Table 1. 867 patients without antiemetic use and 100 (10.3%) patients with at least one antiemetic administration after surgery. Compared to the non-antiemetic use patients, antiemetic use was more common among patients who were young, female, non-smoking, had higher BMI, or undergoing laparoscopic surgery (all *p* < 0.001), and outcomes included bad sleep and vitality. They also displayed significantly higher RINVR and SPONVSS scores (both *p* < 0.001) than non-antiemetic use patients.

**Table 1:** Patient antiemetic use characteristics

Variable	Total	Antiemetic use		*P*-value
		No	Yes	
**Patient number (N)**	967	867 (89.7%)	100 (10.3%)	
**Baseline characteristics**				
**Age**	53.70 ± 15.57	54.49 ± 15.27	46.87 ± 16.55	**< 0.001**
**Gender**				**0.002**
Male	307 (31.7%)	289 (33.3%)	18 (18.0%)	
Female	660 (68.3%)	578 (66.7%)	82 (82.0%)	
**Kidney function**				
eGFR	84.62 ± 32.22	83.61 ± 28.48	93.38 ± 54.27	0.079
**Body measurement**				
Waist/Hip	0.91 ± 0.08	0.91 ± 0.08	0.90 ± 0.07	0.460
BMI	26.57 ± 6.59	26.08 ± 6.16	30.81 ± 8.42	**< 0.001**
**Other data**				
Diabetes	198 (20.5%)	173 (20.0%)	25 (25.0%)	0.236
Smoking	230 (23.8%)	221 (25.5%)	9 (9.0%)	**< 0.001**
Anesthesia time	186.71 ± 90.89	187.87 ± 92.51	176.61 ± 75.07	0.241
**Surgery method**				**< 0.001**
Upper abdominal	129 (13.3%)	125 (14.4%)	4 (4.0%)	
Lower abdominal	89 (9.2%)	85 (9.8%)	4 (4.0%)	
Laparoscopic surgery	210 (21.7%)	157 (18.1%)	53 (53.0%)	
VAT	148 (15.3%)	130 (15.0%)	18 (18.0%)	
Other ^a^	391 (40.4%)	370 (42.7%)	21 (21.0%)	
**Pain**				
NRS rest	2.33 ± 1.39	2.32 ± 1.40	2.41 ± 1.27	0.573
NRS move	4.62 ± 1.73	4.64 ± 1.75	4.51 ± 1.51	0.465
NRS cough	5.48 ± 1.82	5.51 ± 1.85	5.24 ± 1.52	0.096
**Outcome information**				
Bad sleep	532 (55.0%)	459 (52.9%)	73 (73.0%)	**< 0.001**
Bad vitality	172 (17.8%)	141 (16.3%)	31 (31.0%)	**< 0.001**
Bad sleep and vitality	132 (13.7%)	104 (12.0%)	28 (28.0%)	**< 0.001**
**PONV Score**				
RINVR	4.83 ± 7.57	3.34 ± 6.04	17.72 ± 7.26	**< 0.001**
SPONVSS	1.82 ± 3.02	1.22 ± 2.37	6.98 ± 3.15	**< 0.001**

^a^ Other methods include thyroid cancer surgery, breast surgery, orthopedic surgery, and spine surgery

eGFR: estimated Glomerular filtration rate, VAT: Video-assisted thoracotomy, NRS: Numerical rating scale, BMI: body mass index, eGFR: estimated glomerular filtration rate, SPONVSS: simplified postoperative nausea and vomiting severity score (0–12)

A ROC curve analysis of RINVR was performed to obtain the area under curve (AUC: 0.920, 95% CI: 0.893–0.946). All 18 combinations had similar AUC on antiemetic prescription (AUC range 0.915–0.922; *p* < 0.001); bad sleep (AUC range 0.614–0.617; *p* < 0.001); bad vitality (AUC range 0.623–0.629; *p* < 0.001) and both bad sleep and vitality (AUC range 0.662–0.668; *p* < 0.001) (Table 2). We selected vomiting times, retching times, and nausea frequency from the RINVR (Table S1 and Table S2) for our SPONVSS because this combination showed the highest overall AUC for predicting antiemesis use, sleep quality, vitality, and bad sleep and vitality (Table S3). The AUC for antiemesis usage with SPONVSS was similar to RINVR (0.919, 95% CI: 0.893–0.946), but higher than the incidence of PONV (IPONV) (0.816, 95% CI: 0.784–0.848) (Table 2). The optimal cut-off value was 3 (out of 12), with a sensitivity of 0.910 and a specificity of 0.830.

**Table 2:** The area under the curve (AUC) of Rhodes index of nausea retching and vomiting score, the incidence of PONV, and 18 combinations simplified from a selection of one element from each phase of RINVR

	Antiemesis					Sleep Bad					Vitality Bad					BSV			
	AUC	S.E.	P1	P2		AUC	S.E.	P1	P2		AUC	S.E.	P1	P2		AUC	S.E.	P1	P2
RINVR	0.920	0.014	-	**< 0.001**		0.615	0.018	-	**< 0.001**		0.628	0.026	-	**< 0.001**		0.665	0.028	-	**< 0.001**
IPONV	0.816	0.016	**< 0.001**	-		0.589	0.018	**< 0.001**	-		0.591	0.024	**< 0.001**	-		0.617	0.023	**< 0.001**	-
V1N1R1	0.921	0.014	0.626	**< 0.001**		0.617	0.018	0.071	**< 0.001**		0.627	0.025	0.815	**< 0.001**		0.666	0.028	0.728	**< 0.001**
V1N1R2	0.922	0.014	0.402	**< 0.001**		0.616	0.018	0.229	**< 0.001**		0.626	0.025	0.344	**< 0.001**		0.665	0.028	0.744	**< 0.001**
V1N2R1	0.919	0.014	0.832	**< 0.001**		0.616	0.018	0.133	**< 0.001**		0.629	0.026	0.254	**< 0.001**		0.668	0.028	0.118	**< 0.001**
V1N2R2	0.920	0.014	0.977	**< 0.001**		0.616	0.018	0.430	**< 0.001**		0.628	0.026	0.733	**< 0.001**		0.667	0.028	0.404	**< 0.001**
V1N3R1	0.921	0.014	0.443	**< 0.001**		0.616	0.018	0.138	**< 0.001**		0.629	0.026	0.328	**< 0.001**		0.668	0.028	0.229	**< 0.001**
V1N3R2	0.921	0.014	0.436	**< 0.001**		0.616	0.018	0.512	**< 0.001**		0.628	0.025	0.907	**< 0.001**		0.666	0.028	0.707	**< 0.001**
V2N1R1	0.918	0.014	0.445	**< 0.001**		0.616	0.018	0.355	**< 0.001**		0.624	0.025	**0.030**	**< 0.001**		0.661	0.028	**0.035**	**< 0.001**
V2N1R2	0.919	0.014	0.534	**< 0.001**		0.615	0.018	0.928	**< 0.001**		0.623	0.025	**0.003**	**< 0.001**		0.660	0.028	**0.004**	**< 0.001**
V2N2R1	0.914	0.014	**0.026**	**< 0.001**		0.615	0.018	0.978	**< 0.001**		0.627	0.026	0.584	**< 0.001**		0.664	0.028	0.441	**< 0.001**
V2N2R2	0.915	0.014	**0.041**	**< 0.001**		0.614	0.018	0.435	**< 0.001**		0.626	0.025	0.207	**< 0.001**		0.663	0.028	0.155	**< 0.001**
V2N3R1	0.916	0.014	0.163	**< 0.001**		0.615	0.018	0.913	**< 0.001**		0.627	0.025	0.643	**< 0.001**		0.664	0.028	0.400	**< 0.001**
V2N3R2	0.916	0.014	0.187	**< 0.001**		0.614	0.018	0.314	**< 0.001**		0.625	0.025	0.149	**< 0.001**		0.662	0.028	0.080	**< 0.001**
V3N1R1	0.918	0.014	0.386	**< 0.001**		0.615	0.018	0.992	**< 0.001**		0.627	0.025	0.515	**< 0.001**		0.663	0.028	0.235	**< 0.001**
V3N1R2	0.919	0.014	0.565	**< 0.001**		0.614	0.018	0.369	**< 0.001**		0.626	0.025	0.131	**< 0.001**		0.662	0.028	0.050	**< 0.001**
V3N2R1	0.915	0.014	**0.019**	**< 0.001**		0.614	0.018	0.446	**< 0.001**		0.629	0.026	0.349	**< 0.001**		0.666	0.028	0.821	**< 0.001**
V3N2R2	0.915	0.014	**0.029**	**< 0.001**		0.614	0.018	0.148	**< 0.001**		0.628	0.026	0.862	**< 0.001**		0.665	0.028	0.635	**< 0.001**
V3N3R1	0.916	0.014	0.122	**< 0.001**		0.614	0.018	0.372	**< 0.001**		0.629	0.026	0.361	**< 0.001**		0.665	0.028	0.939	**< 0.001**
V3N3R2	0.917	0.014	0.192	**< 0.001**		0.613	0.018	0.090	**< 0.001**		0.627	0.026	0.844	**< 0.001**		0.664	0.028	0.334	**< 0.001**

The linkage of codename and RINVR questionnaire is shown in Table S1. Basically, V1: vomiting times, V2: vomiting volume, V3 vomiting distress, N1: nausea frequency, N2: nausea times, N3: distress from nausea, R1: retch times and R2: distress from retching. RINVR: Rhode index of nausea vomiting and retching, IPONV: incidence of PONV, BSV: both bad sleep and vitality

Furthermore, we detected the simplified postoperative nausea and vomiting severe score and outcomes in Table 3. Antiemesis was administered on 38.2% (91/238) of patients with severe PONV (SPONVSS ≥ 3), whereas 1.2% of the patients with SPONVSS < 3 received rescued antiemetics (*p* < 0.001) (Table 3). To identify the severity of PONV associated with the postoperative use of antiemetic drugs, univariate and multivariate logistic regression analyses were conducted between variables and antiemetic use (Table 4). We observed the clinical association between simplified PONV severity score and antiemetic use. Univariate logistic regression analysis revealed that RINVR, IPONV, SPONVSS, age, male, BMI, eGFR, and surgery method were significantly associated with antiemetic use. Multivariate logistic regression analysis indicated that antiemetic users had significantly higher odds on SPONVSS (an OR: 1.610, 95% CI: 1.479–1.753) as compared to non-antiemetic users. A significantly higher need for antiemetics treatment was also found among patients who received laparoscopy (an OR: 5.475, 95% CI: 1.528–19.615) and thoracic surgery (an OR: 8.109, 95% CI: 2.311–28.460). It was also found that age, gender, BMI, and eGFR were not predictors for antiemetic use in the multivariate regression model.

**Table 3:** The simplified postoperative nausea and vomiting severe score and outcomes

	SPONVSS ≧ 3		*P* value
	No (*n* = 729)	Yes (*n* = 238)	
Antiemetics use	9 (1.2%)	91 (38.2%)	**< 0.001**
Sleep quality			**< 0.001**
Bad	355 (48.7%)	177 (74.4%)	
OK	251 (34.4%)	42 (17.6%)	
Good	123 (16.9%)	19 (8.0%)	
Vitality			**< 0.001**
Bad	98 (13.4%)	74 (47.4%)	
OK	467 (64.1%)	141 (59.2%)	
Good	164 (22.5%)	23 (9.7%)	
Bad sleep and vitality	66 (9.1%)	66 (27.1%)	**< 0.001**

SPONVSS: simplified postoperative nausea and vomiting severity scale

*P* < 0.05 means statistically significant

**Table 4:** The clinical association between simplified PONV severity score and antiemetic use

	Antiemesis use (Ref = No)	
	Univariate OR (95% CI)	Multivariate OR (95% CI)
**SPONVSS**	**1.592 (1.484–1.707)**	**1.610 (1.479–1.753)**
**Age**	**0.969 (0.955–0.982)**	1.012 (0.991–1.033)
**Gender**		
Male	**0.439 (0.259–0.745)**	0.834 (0.424–1.640)
Female	Reference	Reference
**Waist hip ratio**	0.368 (0.026–5.217)	
**BMI**	**1.088 (1.060–1.117)**	0.984 (0.942–1.028)
**eGFR**	**1.007 (1.002–1.013)**	1.004 (0.997–1.011)
**Other baseline data**		
Diabetes	1.337 (0.826–2.166)	-
Smoking	0.289 (0.143–0.583)	-
Anesthesia time	0.999 (0.996–1.001)	-
**Surgery method**		
Upper abdomen	Reference	Reference
Lower abdomen	1.471 (0.358–6.042)	2.031 (0.407–10.145)
Laparoscope	**10.549 (3.717–29.939)**	**5.475 (1.528–19.615)**
VAT	**4.327 (1.425–13.141)**	**8.109 (2.311–28.460)**
Other ^a^	1.774 (0.597–5.266)	3.044 (0.896–10.348)
**Pain**		
NRS rest	1.044 (0.900–1.210)	-
NRS move	0.956 (0.847–1.079)	-
NRS cough	0.918 (0.817–1.032)	-

^a^ Other methods include thyroid cancer surgery, breast surgery, orthopedic surgery, and spine surgery

VAT: Video-assisted thoracotomy, NRS: Numerical rating scale, BMI: body mass index, eGFR: estimated glomerular filtration rate, SPONVSS: simplified postoperative nausea and vomiting severity score (0–12)

Of note, in this study, routine electrocardiogram (ECG) monitoring was not conducted. However, all patients were evaluated daily through face-to-face interviews by research assistants, who inquired about any new-onset serious arrhythmias or medical emergencies. No cases of arrhythmias necessitating emergency intervention or transfer to the intensive care unit were reported. Only one patient developed new-onset atrial fibrillation postoperatively.

### SPONVSS and sleep quality

Characteristics of the population according to sleep status and analyses of the risk factors for sleep status are shown in Table 5. In terms of sleep quality after surgery, 825 patients (85.3%) did not have good sleep after the operation either due to difficulty falling asleep (“Bad” sleep quality) or feeling rested with partial sleep (“Okay” sleep quality) (Table 5). Among these patients, 67.3% were female, 20.1% had diabetes, and 23.9% had a smoking habit. The mean age, eGFR, anesthesia time, and SPONVSS of these patients were 54.24 ± 15.32 years, 84.05 ± 29.38 mL/min/1.73m^2^, 190.81 ± 92.14 min, and 2.00 ± 3.17, respectively. In terms of SPONVSS, sleep quality (Good vs. Bad + OK, *p* < 0.05) differed significantly in age, anesthesia time, surgery method, NRS score of rest, moving, and coughing. To correlate sleep status with its risk factors, univariate and multivariate logistic regression analyses were performed between variables and sleep quality (Table 5). Univariate logistic regression analysis showed that sleep quality is significantly associated with SPONVSS score, age, anesthesia time, and NRS scores of patients at rest, moving, and coughing. Multivariate logistic regression analysis revealed that bad sleep quality had a significantly higher correlation with SPONVSS (OR: 1.222, 95% CI: 1.111–1.344), age (OR: 1.017, 95% CI: 1.004–1.030), and NRS score of the rest (OR: 1.504, 95% CI: 1.242–1.823) surgery method. Anesthesia time, and NRS scores when moving or coughing were not found to be independent predictors of sleep quality. Good sleep quality was only found in 8.0% of the patients with severe PONV (SPONVSS ≥ 3) and in 16.9% of patients with SPONVSS < 3, with an Odd ratio of 0.427 (95% CI: 0.257–0.710) (Table 3).

**Table 5:** Characteristics of population according to sleep status and analyses of the risk factors for sleep status

	Sleep		*P*-value	Sleep (Ref = good)	
	Bad or okay (*n* = 825)	Good (*n* = 142)		Univariate OR (95% CI)	Multivariate aOR (95% CI)
**SPONVSS**	2.00 ± 3.17	0.78 ± 1.58	**< 0.001**	**1.226 (1.114–1.349)**	**1.222 (1.111–1.344)**
**Age**	54.24 ± 15.32	50.61 ± 16.67	**0.016**	**1.015 (1.004–1.027)**	**1.017 (1.004–1.030)**
**Gender**			0.115		
Male	270 (32.7%)	37 (26.1%)		1.381 (0.924–2.064)	
Female	555 (67.3%)	105 (73.9%)		Ref	Ref
**Waist/Hip**	0.91 ± 0.08	0.90 ± 0.08	0.803	1.336 (0.137–12.990)	
**BMI**	26.55 ± 6.64	26.72 ± 6.32	0.775	0.996 (0.970–1.023)	
**eGFR**	84.05 ± 29.38	87.91 ± 45.31	0.187	0.997 (0.992–1.002)	
**Other data**					
Diabetes	166 (20.1%)	32 (22.5%)	0.510	0.866 (0.564–1.330)	
Smoking	197 (23.9%)	33 (23.2%)	0.869	1.036 (0.680–1.579)	
Anesthesia time	190.81 ± 92.14	162.86 ± 79.43	**< 0.001**	**1.004 (1.002–1.007)**	1.002 (1.000-1.005)^a^
**Surgery method**			**0.014**		
Upper abdomen	111 (13.5%)	18 (12.7%)		Ref	Ref
Lower abdomen	81 (9.8%)	8 (5.6%)		1.642 (0.681–3.961)	
Laparoscope	190 (23.0%)	20 (14.1%)		1.541 (0.782–3.036)	
VAT	126 (15.3%)	22 (15.5%)		0.929 (0.474–1.821)	
Other ^b^	317 (38.4%)	74 (52.1%)		0.695 (0.397–1.214)	
**Pain**					
NRS rest	2.45 ± 1.36	1.64 ± 1.35	**< 0.001**	**1.596 (1.384–1.841)**	**1.504 (1.242–1.823)**
NRS move	4.73 ± 1.74	4.00 ± 1.53	**< 0.001**	**1.300 (1.163–1.453)**	0.995 (0.789–1.255)
NRS cough	5.57 ± 1.82	4.97 ± 1.72	**< 0.001**	**1.213 (1.093–1.346)**	0.966 (0.803–1.162)

^a^P-value = 0.065

^b^ Other methods include thyroid cancer surgery, breast surgery, orthopedic surgery, and spine surgery

VAT: Video-assisted thoracotomy, NRS: Numerical rating scale, BMI: body mass index, eGFR: estimated glomerular filtration rate,

SPONVSS: simplified postoperative nausea and vomiting severity score (0–12)

### SPONVSS and vitality

In regard to overall health condition after the surgery, 780 patients (81%) did not have good vitality status (Table 6), with 67.8% among them female, 21.8% with diabetes, and 23.7% with a smoking habit. The SPONVSS and several patient characteristics revealed significant differences between the two vitality groups (good vs. not good, *P* < 0.05), including age, diabetes, anesthesia time, surgery methods (including thoracic surgery, upper abdominal surgery, lower abdominal surgery, laparoscopy, bariatric and other operations), and NRS scores of rest, moving, and coughing. Univariate logistic regression analysis showed that bad vitality is significantly associated with SPONVSS, age, diabetes, anesthesia time, surgery methods, and NRS score of rest, movement, and coughing. Multivariate logistic regression analysis indicated significantly lower levels of vitality in relevance to SPONVSS (OR: 1.177, 95% CI: 1.084–1.279), anesthesia time (OR: 1.004, 95% CI: 1.002–1.007), and NRS score of the rest (OR: 1.591, 95% CI: 1.329–1.905). Age, diabetes, surgery methods, and NRS score of move and cough are not predictors of vitality in the multivariate regression model. Good vitality is found in 9.7% of the patients with severe PONV (SPONVSS ≥ 3) and in 22.5% of those with SPONVSS < 3, with an odd ratio of 0.369 (95% CI: 0.828 − 0.536) (Table 3).

**Table 6:** Characteristics of population according to vitality status and analyses of the risk factors for vitality status

	Vitality		*P*-value	(Ref = good)	
	Bad or ok (*n* = 780)	Good (*n* = 187)		Univariate OR (95% CI)	Multivariate OR (95% CI)
**SPONVSS**	2.04 ± 3.21	0.88 ± 1.81	**< 0.001**	**1.196 (1.106–1.293)**	**1.177 (1.084–1.279)**
**Age**	54.34 ± 15.71	51.05 ± 14.72	**0.009**	**1.014 (1.003–1.024)**	1.012 (0.999–1.025)
**Gender**			0.556		
Male	251 (32.2%)	56 (29.9%)		1.110 (0.784–1.571)	
Female	529 (67.8%)	131 (70.1%)		Ref	Ref
**Waist hip ratio**	0.91 ± 0.08	0.90 ± 0.08	0.308	2.878 (0.375–22.417)	
**BMI**	26.60 ± 6.79	26.48 ± 5.67	0.802	1.003 (0.979–1.028)	
**eGFR**	84.58 ± 34.06	84.79 ± 23.06	0.919	1.000 (0.995–1.005)	
**Other data**					
Diabetes	170 (21.8%)	28 (15.0%)	**0.038**	**1.583 (1.023–2.448)**	1.059 (0.656–1.709)
Smoking	185 (23.7%)	45 (24.1%)	0.920	0.981 (0.675–1.426)	
Anesthesia time	193.75 ± 94.65	157.33 ± 65.65	**< 0.001**	**1.006 (1.004–1.008)**	**1.004 (1.002–1.007)**
**Surgery method**			**< 0.001**		
Upper abdomen	112 (14.4%)	17 (9.1%)		Ref	Ref
Lower abdomen	81 (10.4%)	8 (4.3%)		1.537 (0.633–3.733)	1.628 (0.648–4.089)
Laparoscope	180 (23.1%)	30 (16.0%)		0.911 (0.480–1.727)	1.052 (0.512–2.160)
VAT	128 (16.4%)	20 (10.7%)		0.971 (0.485–1.946)	1.342 (0.640–2.815)
Other ^a^	279 (35.8%)	112 (59.9%)		**0.378 (0.217–0.659)**	0.623 (0.334–1.163)
**Pain**					
NRS rest	2.50 ± 1.26	1.62 ± 1.65	**< 0.001**	**1.684 (1.476–1.922)**	**1.591 (1.329–1.905)**
NRS move	4.78 ± 1.63	3.98 ± 1.96	**< 0.001**	**1.339 (1.209–1.483)**	0.981 (0.792–1.215)
NRS cough	5.61 ± 1.72	4.96 ± 2.08	**< 0.001**	**1.231 (1.120–1.353)**	0.891 (0.750–1.058)

^a^ Other methods include thyroid cancer surgery, breast surgery, orthopedic surgery, and spine surgery

VAT: Video-assisted thoracotomy, NRS: Numerical rating scale, BMI: body mass index, eGFR: estimated glomerular filtration rate, SPONVSS: simplified postoperative nausea vomiting severity scale

### SPONVSS and its association with bad sleep and bad vitality (BSV) quality

For the functional outcomes after surgery, 132 (13.6%) patients are found to have both bad sleep quality and bad vitality (BSV). Among these patients, 71.2% were female; 24.2% had diabetes, and 19.7% had a smoking habit. SPONVSS and several patient characteristics reveal significant differences between the BSV groups (good or not so bad vs. bad, *P* < 0.05), including anesthesia time, surgery methods, and NRS score of rest, move, and cough. Univariate logistic regression analysis shows that BSV is significantly associated with SPONVSS, anesthesia time, methods of surgery, and NRS score of rest, moving, and coughing (Table S4). Multivariate logistic regression analysis indicates that BSV is significantly related to SPONVSS (OR: 1.267, 95% CI: 1.191–1.348) and NRS score of moving (OR: 1.318, 95% CI: 1.002–1.733). BSV occurred in 27.7% of the patients with SPONVSS ≥ 3 and 9.1% of those with SPONVSS < 3, with an odd ratio of 3.855 (95% CI: 2.636–5.638) (Table S4).

### SPONVSS is associated with simplified apfel score

The ratio of SPONVSS more than 3 was 1.3, 7.1, 20.6, 39.1, and 31.9% for an Apfel score of 0–4, respectively. The ROC between SPONVSS and the simplified Apfel score is 0.648. The sensitivity and specificity of an Apfel score ≥ 3 to identify SPONVSS were 71.0 and 48.1%, respectively. The odd ratio between severe PONV (SPONVSS ≥ 3) and simplified Apfel score ≥ 3 is 2.638 (95% CI = 1.924–3.616).

Supplementary Table 1 summarizes the proportion of patients receiving prophylactic treatment for PONV according to risk level.

## Discussion

This study has developed an easy-to-use measurement, SPONVSS, by simplifying RINVR after validating it as instrumental for measuring PONV severity by relating it to whether antiemetics were prescribed to the participants. Our results show that the SPONVSS values show a similar impact on antiemetic prescription to those of RINVR, both with AUCs higher than that of IPONV. The Multivariate logistic regression analyses suggest that, in addition to the NRS pain intensity, SPONVSS is an independent risk factor of bad sleep status and lower levels of vitality [25]. While RINVR is mainly applied to measure chemotherapy-induced nausea and vomiting in cancer patients, RINVR and SPONVSS are found to be also useful in quantifying PONV intensity for surgical patients. The patients with a SPONVSS < 3 have a lower demand for rescued antiemetics, better sleep, and vitality when interviewed the day after surgery. We have validated the SPONVSS can be used as the PONV severity score. The quantized SPONVSS simply measured nausea, vomiting, and retch times with Likert scale (0–4) is highly related to anti-emetics prescribed representing it can be used to monitor the PONV prevention and treatment effects. It is useful as a quality indicator for post-operation care improvement.

This study proves that the intensity of PONV, as well as pain intensity, is an independent factor for sleep quality and vitality after surgery. PONV is often considered a minor issue because, like wound pain, it typically decreases with time and usually lasts only 24 h. As a result, it is not regarded as an issue to be dealt with immediately after an operation like a wound pain. Moreover, the debilitating impact of PONV on patients is less easily quantified and few studies have investigated this issue regarding patient-perceived limitations in physical activity, concentration, appetite, and sleep, during the early postoperative recovery [26]. Here we found that severe PONV (SPONVSS ≥ 3) besides postoperative pain is an independent risk factor for poor sleep quality and vitality. Clinically, many patients with PONV experience requested anesthesiologists to provide PONV-free anesthesia. We also found that severe PONV can be recalled 3 months after operation [25]. By contrast, almost all patients who reported good sleep and vitality were free from severe PONV. Therefore, aggressively managing PONV may not only decrease PONV itself but may improve sleep and vitality quality.

Several considerations drawn from existing PONV instruments have prompted the use of vomiting time, retching times, and nausea duration to be used as the elements of SPONVSS (Table 2). First of all, all the combinations of the RINVR elements have a strong correlation with antiemetic use (AUC 0.915–0.922), bad sleep (AUC 0.660–0.668), and bad vitality (AUC 0.613–0.617) (Table 2). The reduction of the number of elements and their clear definitions may facilitate implementation. The need for simplicity also explains the fact that both simplified PONV impact score [27] and SPONVSS use vomiting times and retching times. In terms of nausea, however, Myles *et al.s’* detailed descriptions of how long nausea affects a patient’s daily life [2], though comprehensive and thorough, are somewhat subjective and may present challenges in practical application, making the scale more complex to implement. Though we selected vomiting and retching times and nausea frequency as the SPONVSS elements, it is possible to study PONV severity with alternative combinations of nausea, vomiting, and retching dimensions.

Our results show that 61% of the patients with PONV have SPONVSS ≥ 3 (3/12) as opposed to 18% of the patients with PONV who had SPONVIS ≥ 5 (5/6) in Myles et al. study (Table 3). The high correlation between our SPONVSS and rescue antiemetics prescription aligns well with the experiences of doctors, nurses, caregivers, and patients, indicating broad applicability. Besides, we could not follow SPONVIS because our study design came earlier than the relevant reports. With the measurement of SPONVSS, a more aggressive or preventive antiemetics use will hopefully promote postoperative recovery not only in PONV but also in sleep quality and vitality, as shown in Table 4.

The Apfel score has moderate power on the prediction of SPONVSS. PONV has multiple factorials to develop personalized precision medicine, to manage the PONV to improve the prediction power and rescue on the severe PONV on the different operation or anesthesia methods is prompt [28]. The pilot analysis shows there is a good correlation between the SPONVSS to the simplified Apfel score. Further study will be performed to find out the risk factors of SPONVSS to improve prophylactic dosing the antiemesis.

In this study, we observed that patients with diabetes had significantly lower postoperative vitality compared to those without diabetes. This finding aligns with the existing literature, which highlights that a sense of vitality measured by single items in the quality of life questionnaire short form 36 (SF36) is associated with cardiovascular events in type 2 diabetes independently of traditional risk factors and arterial stiffness [29]. Additionally, the presence of comorbidities significantly reduces vitality by impairing both physical and mental health dimensions. Mental health comorbidities, in particular, play a significant role in deteriorating quality of life and increasing mortality risks [30]. Therefore, addressing comorbidities comprehensively, including mental health aspects, is essential for improving postoperative vitality and overall recovery in surgical patients.

### Limitations

The data were subjective ratings of sleep quality and overall vitality reported by patients and taken down by research assistants without quantitative scoring. Additionally, the data on the specific types and dosages of anesthetic drugs used were not comprehensively detailed, which can affect the incidence of PONV. The other limitation of this study is the assessment of PONV using RINV after anesthesia remains less common in real-world studies. Nevertheless, our current study has confirmed this application besides another study in Korea [31]. What remains to be determined is the cutting point for clinically important PONV with the scores of classical RINV or simplified RINV. Based on current findings, we anticipate better management of clinically important PONV assessed by the SPONVSS method will reduce the severity of postoperative emetic response, and improve sleep quality and vitality for better recovery after surgery (Tables 5 and 6). Additionally, this study’s evaluation of sleep quality post-surgery was primarily focused on the impact of PONV and PDNV. However, we recognize that numerous other factors can affect sleep quality, including pain, nursing evaluations for vital signs, coughing, incentive spirometry, patient movement, and room temperature. The absence of data on these variables is a limitation of our study and should be considered when interpreting the results. Future studies are recommended to incorporate objective sleep metrics, such as actigraphy, in order to provide more robust and quantifiable sleep assessments, thereby strengthening the overall evidence levels.

## Conclusions

In conclusion, we developed a simple and practical scale to monitor PONV intensity in a broad clinical surgical setting. High SPONVSS scores (≥ 3) are independent risk for rescue antiemesis, poor sleep quality, and vitality. This scale will be useful to monitor postoperative care quality and to improve postoperative care.

## Electronic supplementary material

Below is the link to the electronic supplementary material.

Supplementary Material 1

## Data Availability

All data generated during this study are included in this published article and its supplementary information files.

## Abbreviations

PONV: Postoperative nausea and vomiting
IPONV: Incidence of PONV
RINVR: Rhodes Index of Nausea, Vomiting, and Retching
SPONVSS: Simplified PONV severity score
SPONVIS: Simplified PONV Impact Scale
BMI: Body mass index
ASA: American Society of Anesthesiology
AUC: Area under the curve
BSV: Bad sleep and bad vitality
ROC: Receiver operating characteristic
OR: Odds ratio
Cis: Confidence intervals
NRS: Numeric scale
eGFR: Estimated Glomerular filtration rate
