# Supplementary material for: A prospective cohort observational study to validate a simplified postoperative nausea and vomiting severity scale and its effects on sleep and vitality
Source: BMC Anesthesiol. 2025 May 10;25:236. doi: 10.1186/s12871-025-03074-2 (PMC12065345; doi:10.1186/s12871-025-03074-2)
Supplement: Supplementary file 1 — Supplementary Material 1 [file 12871_2025_3074_MOESM1_ESM.docx]

**Table S1.** The comparison chart of RINVR questionnaire and the codename used in this study.

| codename | RINVR question | RINVR# |
| --- | --- | --- |
| V1 | In the last ( ) hours, I threw up ○○ times. | 1 |
| R2 | In the last ( ) hours, from retching and dry heaves, I felt ○○ distress. | 2 |
| V3 | In the last ( ) hours, from vomiting or throwing up, I felt ○○ distress. | 3 |
| N1 | In the last ( ) hours, I have felt nauseated or sick to my stomach. | 4 |
| N3 | In the last ( ) hours, from nausea/sickness to my stomach, I have felt ○○ distress. | 5 |
| V2 | In the last ( ) hours, each time I threw up, I produced a ○○ amount. | 6 |
| N2 | In the last ( ) hours, I have felt nauseated or sick to my stomach ○○ times. | 7 |
| R1 | In the last ( ) hours, I have had periods of retching or dry heaves without bringing anything up ○○ times. | 8 |

Times grade: No = 0, 1-2 times = 1, 3-4 times = 2, 5-6 times = 3, and > 7 = 4. Frequency grade: Not at all = 0, 1 hour or less = 1, 2-3 hours = 2, 4-6 hours = 3, more than 6 hours = 4.

**Table S2.** Rhodes Index of Nausea, Vomiting, and Retching (RINVR) form.

|  | **4** | **3** | **2** | **1** | **0** | **score** |
| --- | --- | --- | --- | --- | --- | --- |
| **Number of vomiting times** | >7 | 5-6 | 3-4 | 1-2 | 0 |  |
| **Amount of vomiting each time** | >3 | 2-3 | 0.5-2 | 0.5 | 0 |  |
| **Vomiting discomfort level** | Severe | great | moderate | mild | 0 |  |
| **Number of retching times** | >7 | 5-6 | 3-4 | 1-2 | 0 |  |
| **Retching discomfort level** | Severe | great | moderate | mild | 0 |  |
| **Nausea frequency** | >7 | 5-6 | 3-4 | 1-2 | 0 |  |
| **Duration of nausea (hours)** | >6 | 4-6 | 2-3 | <=1 | 0 |  |
| **Nausea discomfort level** | Severe | great | moderate | mild | 0 |  |
| **Total score** |  | | | | |  |

**Table S3.** Simplified PONV severity score (SPONVSS) form.

|  | **4** | **3** | **2** | **1** | **0** | **score** |
| --- | --- | --- | --- | --- | --- | --- |
| **Number of vomiting times** | >7 | 5-6 | 3-4 | 1-2 | 0 |  |
| **Number of retching times** | >7 | 5-6 | 3-4 | 1-2 | 0 |  |
| **Nausea frequency** | >7 | 5-6 | 3-4 | 1-2 | 0 |  |
| **Total score** |  | | | | |  |

| **Table S4.** Characteristics of the population according to bad sleep and vitality status and analyses of the risk factors for BSV status. | | | | | |
| --- | --- | --- | --- | --- | --- |
|  | | | | | |
|  | **BSV** | | **P-value** | **BSV (Ref= good)** | |
|  | Bad  (n=132) | Good  (n=835) |  | Univariate  OR (95% CI) | Multivariate  OR (95% CI) |
| **SPONVSS** | 4.00±4.20 | 1.47±2.63 | **< 0.001** | **1.235 (1.174-1.298)** | **1.267 (1.191-1.348)** |
| **Age** | 53.52±17.52 | 53.73±15.25 | 0.892 | 0.999 (0.987-1.011) |  |
| **Gender** |  |  | 0.432 |  |  |
| Male | 38 (28.8%) | 269 (32.2%) |  | 0.851 (0.568-1.274) |  |
| Female | 94 (71.2%) | 566 (67.8%) |  | Ref | Ref |
| **Waist hip ratio** | 0.90±0.07 | 0.91±0.08 | 0.416 | 0.377 (0.036-3.957) |  |
| **BMI** | 27.26±7.77 | 26.46±6.38 | 0.262 | 1.018 (0.991-1.045) |  |
| **eGFR** | 83.96±28.01 | 84.72±32.85 | 0.801 | 0.999 (0.993-1.005) |  |
| **Other data** |  |  |  |  |  |
| Diabetes | 32 (24.2%) | 166 (19.9%) | 0.248 | 1.290 (0.837-1.988) |  |
| Smoking | 26 (19.7%) | 204 (24.4%) | 0.235 | 0.759 (0.480-1.198) |  |
| Anesthesia time | 210.33±102.11 | 182.97±88.48 | **0.001** | **1.003 (1.001-1.005)** | 1.002 (1.000-1.004) ^a^ |
| **Surgery method** |  |  | **< 0.001** |  |  |
| Upper abdomen | 23 (17.4%) | 106 (12.7%) |  | Ref | Ref |
| Lower abdomen | 18 (13.6%) | 71 (8.5%) |  | 1.168 (0.588-2.320) | 1.324 (0.637-2.750) |
| Laparoscope | 44 (33.3%) | 166 (19.9%) |  | 1.222 (0.698-2.139) | 0.834 (0.436-1.593) |
| VAT | 22 (16.7%) | 126 (15.1%) |  | 0.805 (0.425-1.525) | 0.928 (0.465-1.852) |
| Other | 25 (18.9%) | 366 (43.8%) |  | **0.315 (0.172-0.577)** | 0.578 (0.299-1.116) |
| **Pain** |  |  |  |  |  |
| NRS rest | 2.99±1.53 | 2.23±1.33 | **< 0.001** | **1.470 (1.286-1.681)** | 0.876 (0.716-1.071) |
| NRS move | 5.77±1.68 | 4.44±1.66 | **< 0.001** | **1.558 (1.394-1.741)** | **1.318 (1.002-1.733)** |
| NRS cough | 6.65±1.68 | 5.30±1.77 | **< 0.001** | **1.491 (1.346-1.652)** | 1.251 (0.988-1.568) |
| ^a^P-value=0.094 | | | | | |

BSV: bad sleep and vitality, VAT: Video assisted thoracotomy, NRS: Numerical rating scale, BMI: body mass index, eGFR: estimated glomerular filtration rate, BSM: Bad sleep and bad vitality, SPONVSS: simplify postoperative nausea and vomiting severity scale
